# Network Topologies Decoding Cervical Cancer

**DOI:** 10.1371/journal.pone.0135183

**Published:** 2015-08-26

**Authors:** Sarika Jalan, Krishna Kanhaiya, Aparna Rai, Obul Reddy Bandapalli, Alok Yadav

**Affiliations:** 1 Centre for Biosciences and Biomedical Engineering, Indian Institute of Technology Indore, Indore, 452017, India; 2 Complex Systems Lab, Discipline of Physics, School of Basic Sciences, Indian Institute of Technology Indore, Indore, 452017, India; 3 Molecular Medicine Partnership Unit, EMBL-University of Heidelberg, Heidelberg, Im Neuenheimer Feld 350, Heidelberg, Germany; University of Maribor, SLOVENIA

## Abstract

According to the GLOBOCAN statistics, cervical cancer is one of the leading causes of death among women worldwide. It is found to be gradually increasing in the younger population, specifically in the developing countries. We analyzed the protein-protein interaction networks of the uterine cervix cells for the normal and disease states. It was found that the disease network was less random than the normal one, providing an insight into the change in complexity of the underlying network in disease state. The study also portrayed that, the disease state has faster signal processing as the diameter of the underlying network was very close to its corresponding random control. This may be a reason for the normal cells to change into malignant state. Further, the analysis revealed VEGFA and IL-6 proteins as the distinctly high degree nodes in the disease network, which are known to manifest a major contribution in promoting cervical cancer. Our analysis, being time proficient and cost effective, provides a direction for developing novel drugs, therapeutic targets and biomarkers by identifying specific interaction patterns, that have structural importance.

## Introduction

Approximately 528,000 new cases of cervical cancer were diagnosed and 266,000 deaths estimated worldwide in 2012 [1, 2]. The incidence is found to be increasing gradually, mainly in the younger population of women [3]. Though, the infection of human papilloma virus (HPV) has an important role in the occurrence of the disease [4], the percentage of women developing this cancer by infection of HPV alone is about 40% [5, 6]. This indicates that some other factors like genetic susceptibility, dietary issues, environment and indecent lifestyle are responsible for the onset of disease [3]. Inspite of huge investments and extensive research in the last few years, the etiology of cervical cancer is still unclear [7]. Further, this neoplasm is an excellent model for studying the mechanisms involved in cancer maintenance, and presents a reliable way to monitor the biological alterations induced by the disease [8].

A previous study by Alsbeih et al., has shown that somatic mutations in PIK3CA, PTEN, TP53, STK11 and KRAS as well as several copy number alterations lead to the pathogenesis of cervical carcinomas [9]. Few comparative screening analyses of diseased smears in regular intervals have helped to diagnose the stage of cancer in the patient in a cost efficient manner, but these analyses could not deliver successful results for patients in advanced stage [10]. Later, studies on the chemokine network have instigated the researchers to target both chemokines and their receptors for therapeutic intervention, either with antibodies or small molecule antagonists [11]. However, both, complexity as well as variations at every stage of the cancer renders designing drug targets very difficult [12, 13]. Systems biology approaches based on network theory have allowed to investigate vast data gathered using -omics technologies (i.e., gen-, transcript-, prote-, and metabol-omics) in a novel way [14]. Biological processes are considered as complex networks of interactions among numerous components of the cell rather than independent interactions involving only a few molecules [15–17]. Earlier studies based on human disease network reveals that various types of cancers are interlinked to each other through number of pathways, which are altered in different diseases [18]. As cancer is a complex disease, the representation of a malignant cell as a protein-protein interaction (PPI) network and its subsequent comparitive analysis with its normal couterpart can provide an insight into the behavior of cancer cells and may lead to the discovery of new biomarkers [19]. In this study, we analyzed the PPI networks of cervix cells of the uterine tissue for normal and disease states and investigated their structural properties. This comprehensive study enabled us to identify differences between the normal and disease conditions. The structural parameters depict some important proteins which are functionally significant in the occurrence of the disease and can be used for drug targets for a more effective treatment of the disease.

## Results

### Structural properties of cancer networks

The total number of proteins and their interacting partners obtained for normal uterine cervix cell had 4481 nodes and 21801 connections, followed by 2636 nodes and 20040 links for cervical cancer datasets. From these datasets, we obtained various connected components referred as networks. Different properties of the normal and disease networks are summarized in Table 1. The first column in Table 1 indicated that the total number of proteins in the disease dataset is less than that of the normal data. A probable reason behind this could be more availability of the normal data in comparison to the disease one. Another probable reason could be that, in the disease state, many pathways are silenced or protein expressions are altered [20]. Also, for the disease state, there may be involvement of new proteins or pathways which might have not been captured by the analyst yet. Next column in Table 1 depicted the average degree (⟨*k*⟩) of the network. The third column denoted the diameter of the network which indicated the ability of a network for signal transduction [21]. A lower diameter of the disease network (DNC and C1) indicated a faster signaling of pathways when compared to the normal state. This is biologically relevant, as disorders in cancer associated proteins promote the adaptability of faster communication in many major cancer related cellular signaling processes [22]. Further, we calculated the average clustering coefficient (⟨*CC*⟩) of all the networks along with the total number of nodes having CC equal to one (*N*_*cc* = 1_) as tabulated in Table 1. The nodes having *CC* = 1 reflected the formation of complete sub-graphs or cliques comprising of the node under consideration. Also, the higher value of ⟨*CC*⟩ implicated the existence of high number of cliques or close to clique structure in the network [21]. Cliques are known as the building blocks of a network, making the underlying system more robust and stable [23, 24], as well as known to be preserved during evolution [25]. Networks having less number of cliques depicted demolition of building blocks, indicating an unstable system which might be leading to the occurrence of disease.

**Table 1:** Network properties of all the real networks.

Network	*N*_*ori*_	*N*	*N*_*c*_	⟨*k*⟩	*D*	⟨*CC*⟩	*N*_*cc* = 1_	*N*_*cc* = 0_
C1	1804	912	9132	20	7	0.31	32	163
C2	1804	724	5707	15	10	0.29	32	136
DNC	831	719	4711	13	8	0.31	37	141
NNC1	2677	694	3724	10	10	0.36	61	229
NNC2	2677	640	2263	7	12	0.27	49	275

The first column (*N*_*ori*_) represented the total number of proteins (nodes) in the normal not common (*NNC*1 and *NNC*2) networks, common (*C*1 and *C*2) and disease not common (*DNC*) networks, collected using various databases (described in result and discussion section), number of proteins in the largest connected cluster (*N*) and connections (*N*_*c*_), the average degree (⟨*k*⟩), diameter (D), average clustering coefficient⟨*CC*⟩, the number of nodes having CC = 1 (*N*_*cc* = 1_) and CC = 0 (*N*_*cc* = 0_) for all the networks.

**Fig 1:**
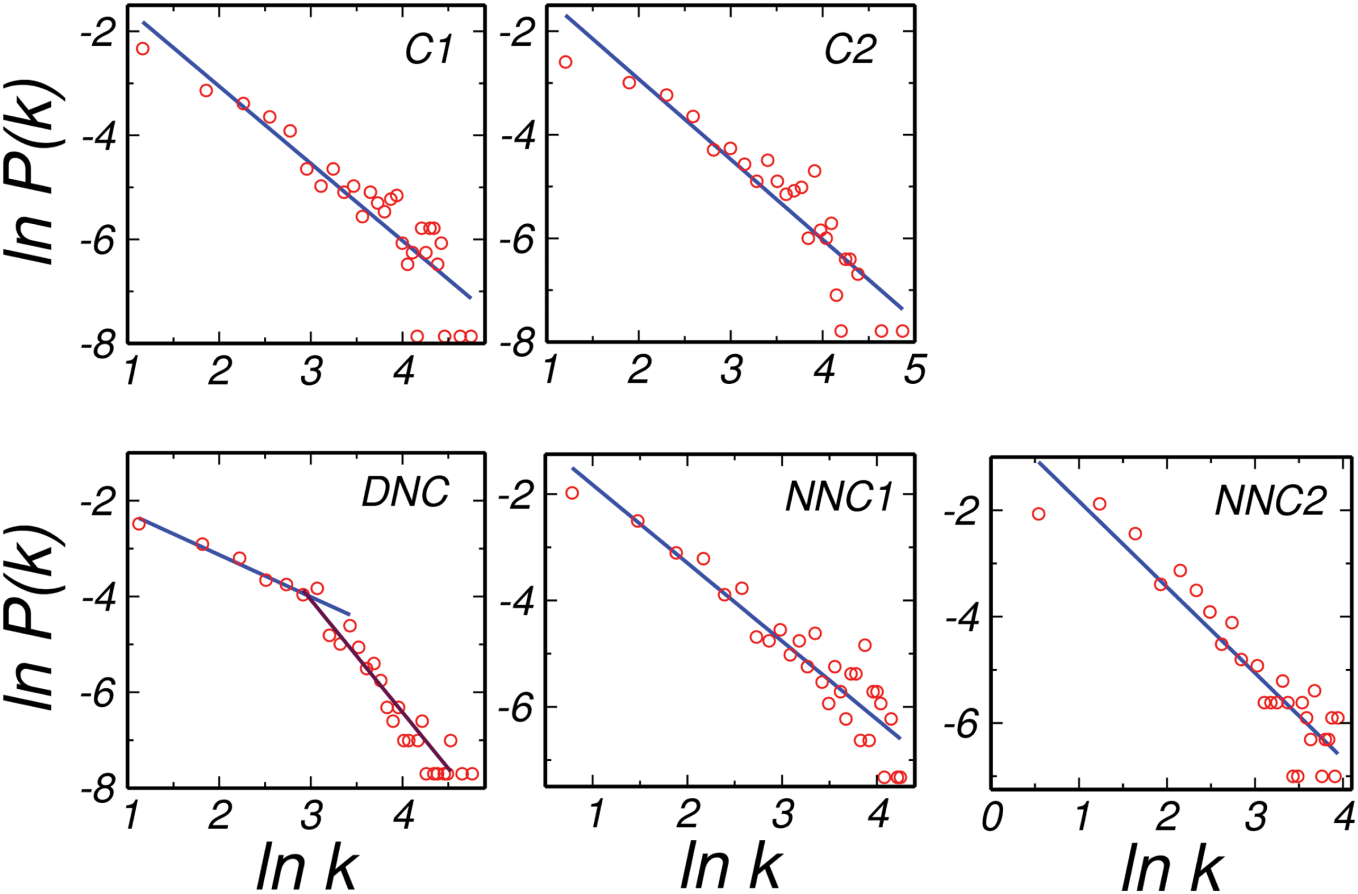
Degree distribution. Degree distribution for *C*1, *C*2, *DNC*, *NNC*1 and *NNC*2 datasets following the power law behavior. Interesting observation is recognised for the DNC network having double power law.

The degree distribution *P*(*k*), of all the networks for both the normal and disease dataset followed power law (Fig 1). This demonstrated that in these networks nodes having very less number of neighbors were in majority coexisting with a few nodes having a very large numbers of interacting partners. Many other biological systems have been known to follow the power law degree distribution with the exponent lying between 2 and 3, suggesting scale free behavior [17]. Another interesting observation was that the DNC network pertained two different power laws as also found in many social and economic systems [26]. This behavior is not found in other networks such as C1, C2, NNC1 and NNC2 and hence made the disease network different. Also, for cervical disease networks, the exponent lied below *two* for all the networks, which may be due to the finite size effect [27].

In order to understand the complexity of interactions as well as to have a deep insight to changes in the interaction patterns of the disease, a comparison of their properties with the corresponding random controls was conducted. Since, the degree sequence is known to be one of the prime features which in turn affect many other properties of a network, random controls were generated using the same degree sequence as of the real networks being investigated here.

### Comparison with random control networks

We compared all the normal and disease networks with the corresponding configuration model which is a random replica of the networks considered here. The configuration model preserves the exact degree sequence of a network [28] by producing random networks for a given degree sequence of an array of size $m=\frac{1}{2}\sum_{i=1}^{N}k_{i}$ which have *random* connections among different elements. We generated ten such realizations for a given degree sequence, various properties of such networks are enlisted in Table 2.

**Table 2:** Network properties of the corresponding configuration model.

Network	⟨*k*⟩	*D*	⟨*CC*⟩	*N*_*cc* = 1_	*N*_*cc* = 0_
C1	20	6	0.11	10	219
C2	16	6	0.09	6	211
DNC	13	6	0.08	5	234
NNC1	11	6	0.08	11	313
NNC2	7	7	0.05	5	376

On comparing all the networks with the corresponding configuration models, we found that the properties of corresponding random controls, which were generated using the same degree sequence as of the real networks, deviated significantly from those of the real networks. Though, the diameter as well as the clustering coefficient of the corresponding random networks were small as expected [21], the interesting part was that, while the diameter of all real networks were much larger than the corresponding random controls, the DNC network had diameter very close to that of the corresponding random model. Since, the diameter of a network is defined as largest of all the shortest paths in a network and reflects the ability of communication among nodes within the network [29], a lower diameter of DNC close to the corresponding randomized network signified that each node of the DNC network was attached to other nodes in very few steps [21]. This further indicated that communication in DNC network was faster in comparison to the other (NNC1 and NNC2) networks [29]. A plausible interpretation for this can be that the signaling of the information in cancer cells was much faster than the normal cells. The faster information flow in DNC may be a reason for improper functioning in the cells leading to the diseased state for example the uncontrolled proliferation of cells. Also, the impact of speedy propagation of signals have already been proven in the case of epileptic seizures, where due to uncontrolled flow of information abnormalities occur [30].

Next, the clustering coefficient of the real networks were much higher than the configuration model (Tables 1 & 2). These higher clustering coefficients indicated that, in the real networks, neighbors were well connected with each other. Also, we found nodes having CC equal to one, which reflected that they are a part of complete sub-graphs (cliques). These cliques are known to provide a clue for disease pathogenesis [31], indicating their importance in cancer networks, a further investigation to these nodes may provide a better understanding of cancer.

**Fig 2:**
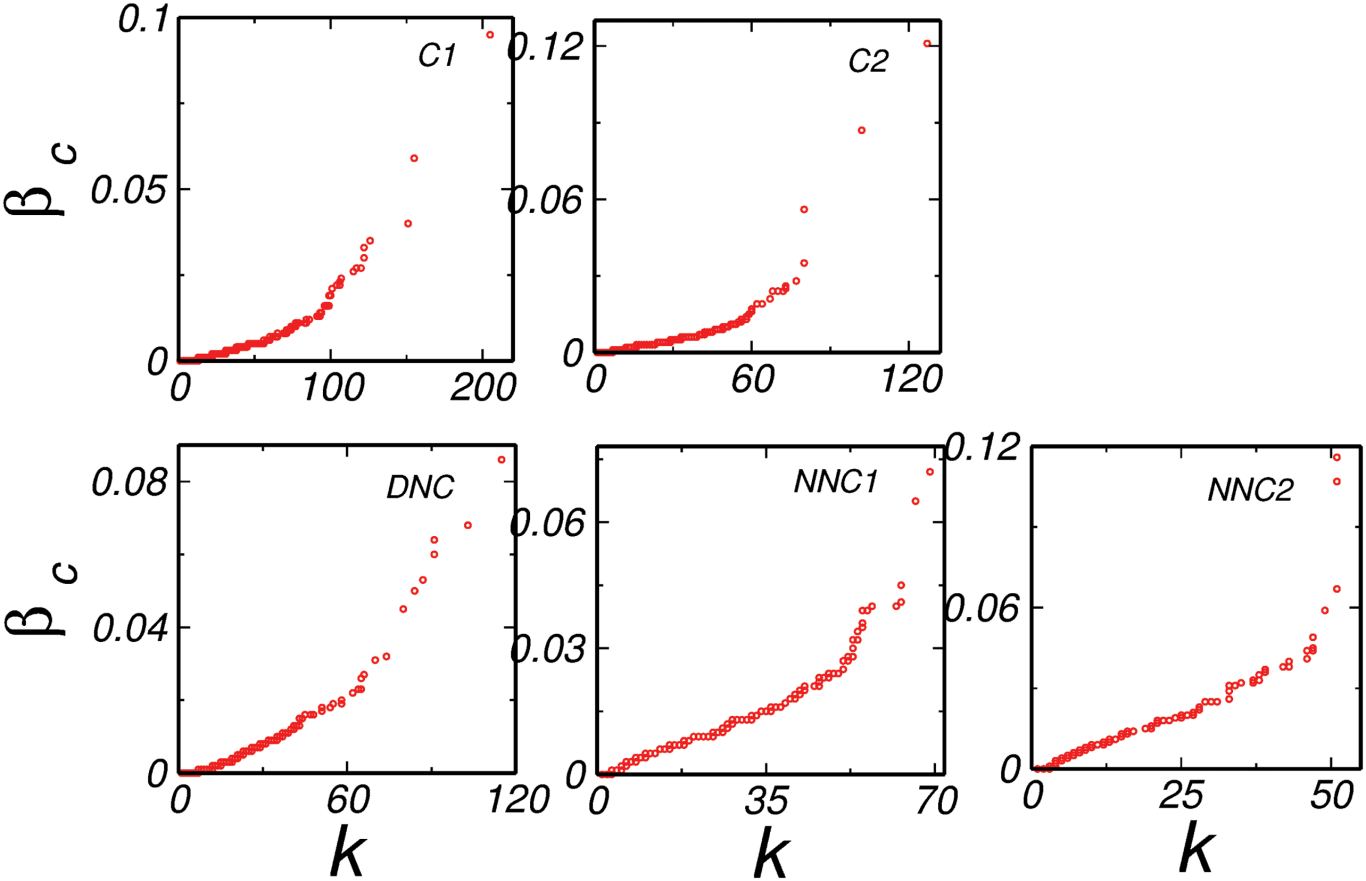
Degree-Betweenness centrality correlation. All the real models common (*C*1 and *C*2), disease not common (*DNC*) and normal not common (*NNC*1 and *NNC*2) network have positive correlation.

**Fig 3:**
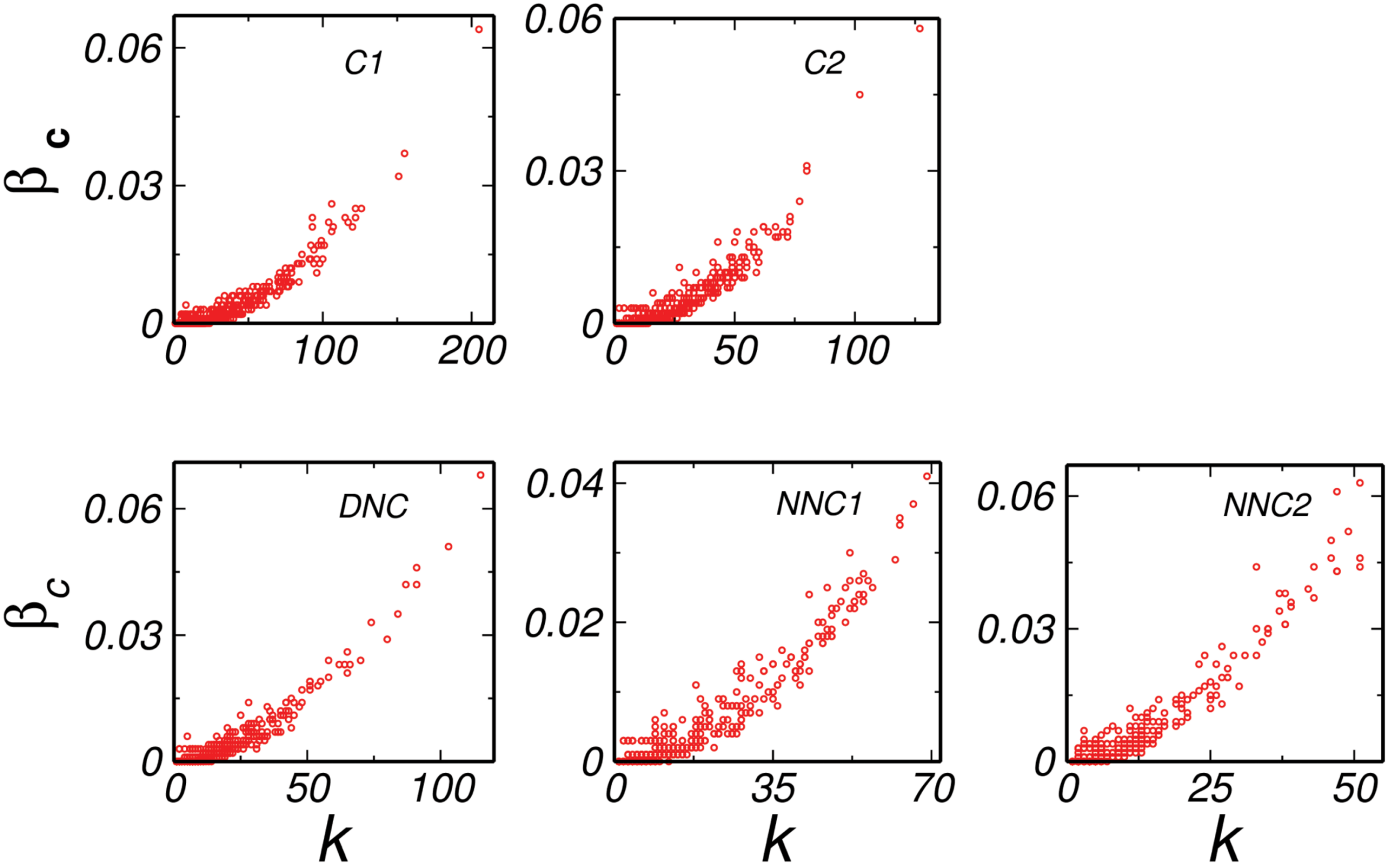
Degree- Betweenness centrality configuration correlation. All the configuration models common (*C*1 and *C*2), disease not common (*DNC*) and normal not common (*NNC*1 and *NNC*2) network shows low betweenness centrality compared to the real model.

Further, it is already reported that the high degree and high betweenness centrality nodes are important since they are found in various pathways in a network [17, 32] and hence we analyzed the degree-betweenness centrality correlation (*k*-*β*_*c*_) for all the networks and compared them with their corresponding random models. We found that, the *k*-*β*_*c*_ for all the common networks depicted an overall positive correlation as expected (Fig 2) because the high degree nodes tend to have more betweenness centrality. This result was similar to their corresponding configuration model (Fig 3). For all the real networks, the highest value of *β*_*c*_ was very high when compared to the corresponding configuration model (Figs 2 and 3). For example, the highest *β*_*c*_ of *C*1 was around 0.094, while the highest *β*_*c*_ of the corresponding configuration model was around 0.063. However, the interesting observation was noted in the DNC network, where the highest value of *β*_*c*_ was as low as that of the corresponding model (0.076 for real and 0.085 for corresponding configuration model). Thus, the node having highest *β*_*c*_ was due to the degree of that node and no additional property was involved. An interesting thing to note that, since corresponding model networks were generated using the same degree sequence as of the real network, the *β*_*c*_ of all the nodes in the network was automatically taken care of according to their degree, and any deviation from it may be arising due to the special position that node possessed due to its functional importance in the network. A lower value of *β*_*c*_ indicated no such preference of a high degree node present in DNC. In order to get a further insight to the difference between the normal and disease states, we diverted our focus to a property which gave us a detailed information about the local behavior of nodes in the network. As discussed earlier, *CC* of a node corresponds to the connectivity between the neighbors of that node, we further analyzed the degree-CC (*k*-*CC*) correlations.

**Fig 4:**
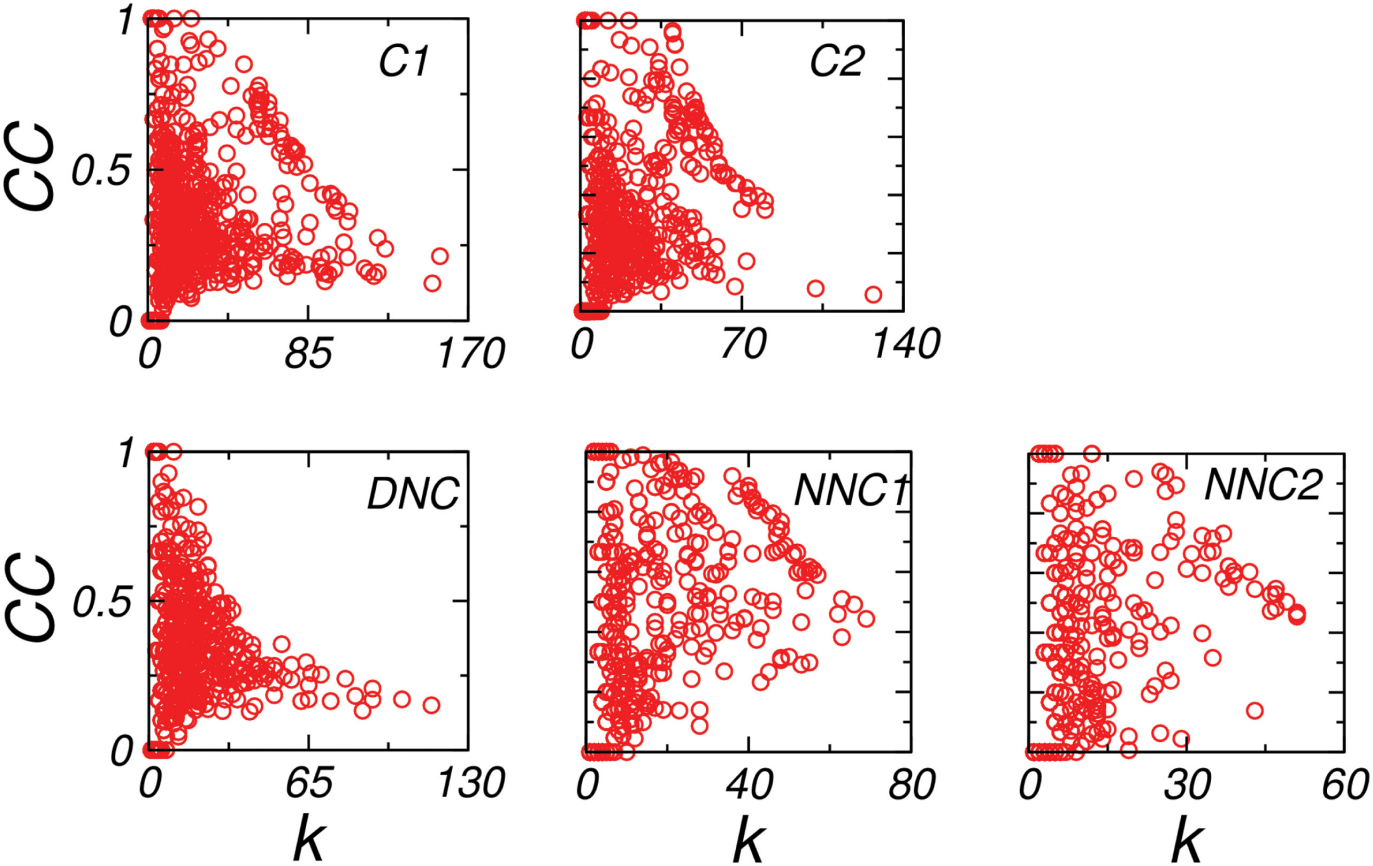
Degree-Clustering coefficient correlation. The figure showed *k*−*CC* correlation of the common (*C*1 and *C*2), disease not common (*DNC*) and normal not common (*NNC*1 and *NNC*2) networks, while the common and DNC networks reflected less random correlation, normal not common exhibited more towards random correlation.

The *k*-*CC* of C1, C2 and DNC networks were overall negatively correlated (Fig 4) as found for other biological systems [17]. However the graph of NNC1 and NNC2 indicated a deviation from this correlation pattern. The NNC1 and NNC2 consisted of a part of interactions yielding an overall negative *k*−*CC* correlation, with major part of interactions being random reflected in absence of any *k*−*CC* correlations. This inturn depicted that the DNC subgraph have an organized pattern [33] whereas, NNC1 and NNC2 deviating from any correlation may be considered more random. This qualitative comparison were done on the basis of their random pattern, as sufficient amount of randomness is an essential ingredient for the functioning of the system [34]. The analysis inferred that the lack of minimum amount of randomness in the network might be a cause of changes from the normal to the disease state by affecting the functional unit of the system (cell) through mutation and alterations in the interactions of proteins.

We further investigated the functional importance of the nodes which were in the high degree regime to understand the biological significance of these nodes in the occurrence of the cervical cancer.

### Functional properties of high degree proteins

**Fig 5:**
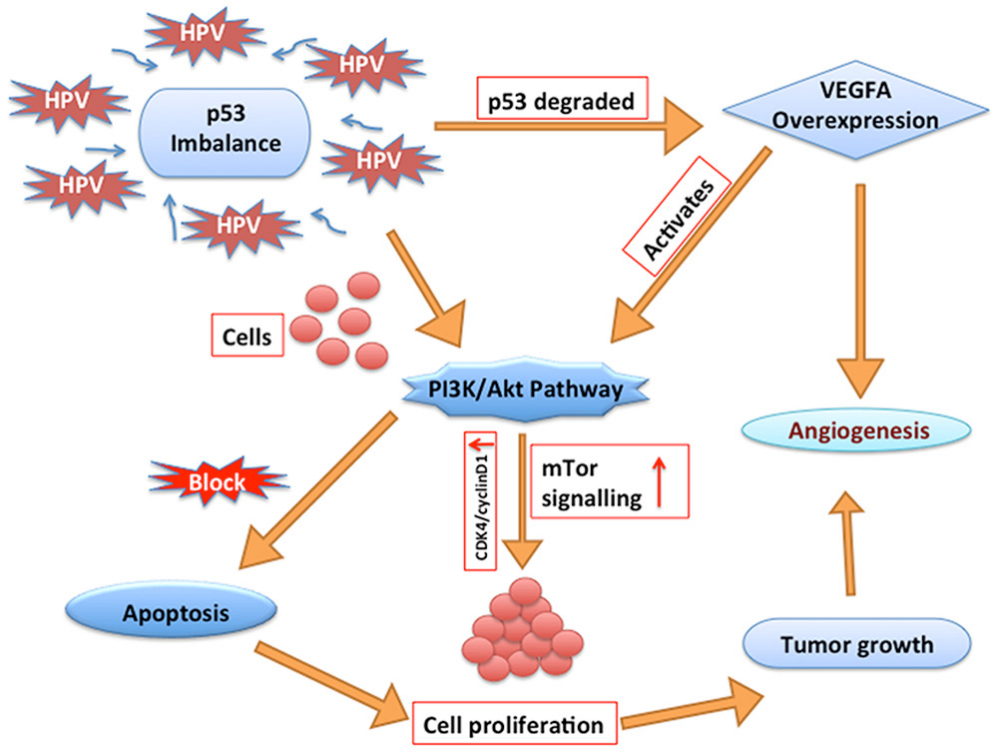
VEGF regulation by p53. The Figure depicted how HPV induced p53 imbalance and regulated the expression of VEGF and PI3K/Akt pathway which in turn leads survival of cells and angiogenesis in cervical cancer.

We determined the degrees of the nodes in all the normal and disease networks and reviewed the functional properties of all the highest degree proteins available from the literature as these nodes are also known to be structurally very important. The proteins (nodes) having the distinctly high degree in disease states were VEGFA, DIF, IL6, PCNA, ESR1, CCND1, TGFB1. All these proteins were known to have distinct roles in cancer development. It was found that over-expression of VEGFA in cervical cell lines increased the tumor growth by activating PI3K/Akt (Fig 5), and subsequently its downstream to the mTor signaling pathway [35]. Furthermore, VEGFA-induced activation of mTor signaling cascades also promoted cancer cell growth through cyclinD1 and CDK4 activation [36]. The invasiveness occurred through MMP2 and MMP3, while inhibition of VEGFA decreased the tumor growth [37]. VEGF-mediated signaling which occurred in tumor cells, contributed to the key aspects of tumorigenesis including the function of cancer stem cells and tumor initiation [38]. Correspondingly clinical studies have verified that VEGF-C expression is closely related to invasion phenotype and affects the patient’s survival in cervical carcinomas [39–41].

**Fig 6:**
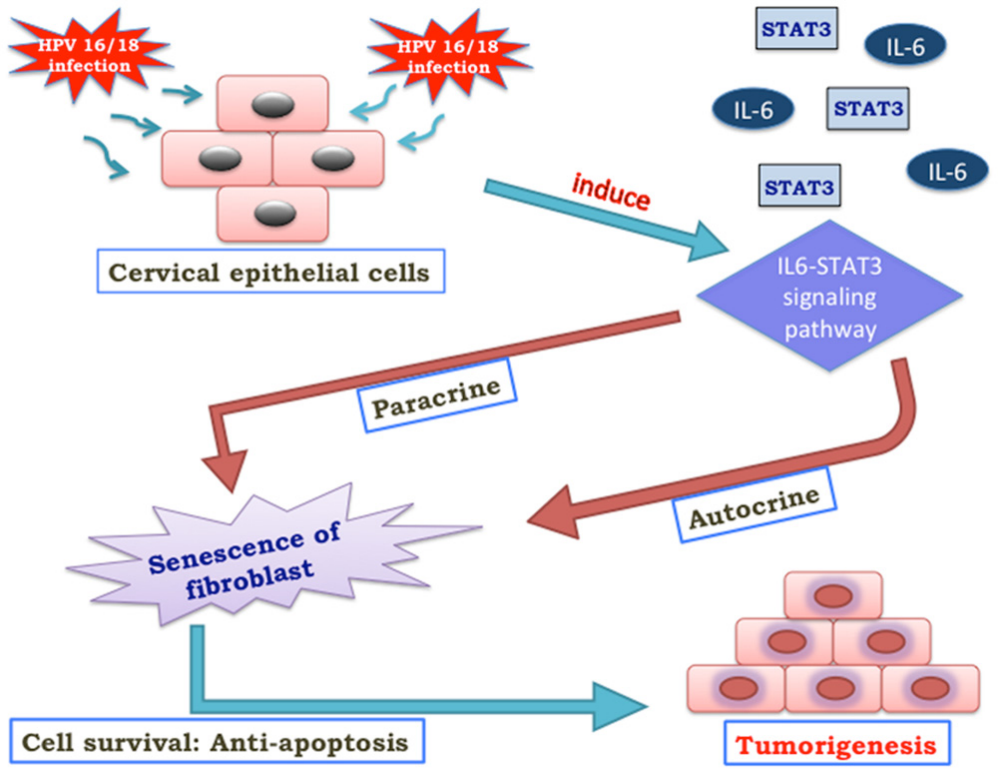
IL-6 regulation. The Figure depicted how IL-6 up-regulated the STAT3 protein by both autocrine and paracrine signaling.

The second protein DIF induced cyclin D1 degradation in cervical cancer cell lines [42] and regulated stalk differentiation. It invaded mainly the mitochondria and localized the mitochondria in HeLa human cervical cancer cells [43]. The next protein in the high degree regime was IL6, whose variation in host immune response by single nucleotide polymorphisms may contribute in cervical cancer risk [44]. Additionally, due to chronic inflammation, IL6 cytokine may increase the risk of developing cervical cancer [45]. It is already known that the cancer-associated fibroblast senescence induced by high-risk -E6, activates IL-6/STAT3 signaling and remodel tumor microenvironment favoring the development of cervical cancer after the elongated latency of the disease. This indicated an option to deprive the activated IL-6/STAT3 network against inflammation including fibroblast senescence in tumor microenvironment (Fig 6) which may be considered as a complement to increase the efficacy of the targeted therapy against HPV 16/18 in cervical cancer [46]. With activated STAT3, IL-6 emerges senescent at early passages in cervical cancer tissues infected with high-risk HPV and activates the STAT3 and cellular senescence [47].

The next top high degree protein PCNA (proliferating cell nuclear antigen) increases due to different grade of CIN (Cervical intra-epithelial neoplasia) lesions in cervical carcinoma. Its positivity was also found to be increased from basal to superficial layers [48] and indicated the chances of cervical carcinoma. As the grade of cervical lesion becomes higher from the normal epithelium to SCC, the expression of PCNA is significantly increased [49]. Up-regulation of PCNA was closely associated with HR-HPV and progressive CIN. However, the fact that PCNA is also expressed in normal squamous epithelium precludes the use of this marker as a potential screening tool for this cancer [50]. The protein having high degree in disease is ESR1 (Estrogen receptor-alpha). Studies revealed that the loss of ESR1 expression has a major role in cervical cancer progression. Methylation of ESR1 was also found in many cervical cancer patients [51]. The next protein CCND1, was found functioning in G to A polymorphism (G870A) of cyclin D1 (CCND1), and changed its spliced transcript. Since transcript b, which was expressed by the CCND1 870A allele lacked PEST motif and critical for the degradation of cyclin D1, this process lead to an over accumulation of cyclin D1 in the cell which promoted increased cell proliferation and was associated with higher tumor grading of cervical cancer patients [52, 53]. The next protein TGFB1 functioned to stimulate apoptosis and inhibit the growth factor. This also increased migration and invasiveness and resulted in metastases. Metastases contributed to the development of different types of cancer [54]. Later, down regulation of SMAD4, which affected the metastatic potentials in early stages of cervical cancer, was associated with TGFB1 down regulation and incriminated cervical cancer [55].

Proteins (nodes) having the highest degree in normal states were CDC6, CCNA2, CENP-F, MKI67IP. Among these, CDC6 played critical roles in DNA replication and carcinogenesis, but biological significance of the CDC6 on cervical carcinogenesis is still unknown [56]. Also, previous research on cervical cancer cases shows that CDC6 protein was preferentially expressed in high grade lesions and in invasive squamous cell carcinoma indicating changes in up-regulation of CDC6 thereby cell proliferation and invasion.[57] The next protein CCNA2 (also known as CyclinA2) was significantly over-expressed in various cancer types, which indicated its potential roles in cancer transformation and progression [58]. However, cervical cancer and relation with this protein has never been reported to our knowledge. Some studies have shown that over expression of cyclin A2 is associated with overall reduced survival [59, 60], while others have reported an association with improved survival [61]. It may be possible that cyclin A2-CDK contributes to tumorigenesis by the phosphorylation of oncoproteins or tumor suppressors like p53 [62]. The protein CENP-F (Centromere protein F) possess significant correlation of the expression of auto-antibodies with cancer and has been described in many studies [63]. It has also been reported that expression of CENP-F is an important predictor among the genes highly expressed in tumors of patients with inferior survival [64]. Next, protein MKI67IP is an rRNA transcription protein which is present in the tumor co-expression network [65]. MKI67IP gene encodes a nucleolar protein that interacts with the forkhead-associated (FHA) domain of the Ki-67 antigen, a proliferation related protein. MKI67IP interacting with FHA might promote tumorogenesis as, FHA domain is a phosphopeptide-binding domain present in a variety of nuclear cellular proteins involved in DNA repair, cell cycle arrest, or pre-mRNA processing [66]. As of our knowledge, no experimental analysis is done to confirm its involvement in tumor formation.

Further, we analyzed the functional role of the proteins common in disease and normal states. The high degree proteins found common in both the normal and disease were TP53, CK1, AKT1, SUMO1, SUMO2, SRSF1 and XRCC3. The first protein TP53 is clearly a component of one of the pathways activated in response to DNA damage [67–71] and its mutations in cervical carcinomas are associated with aggressive cancer and late occurrence in tumor progression [72]. Also p53 can inhibit cell proliferation by blocking entry into the S phase of the cell cycle and is also a master regulator of apoptosis. The expression of p53 is increased proportionally to the grade of CIN and cervical cancer. Therefore, p53 immunoreactivity can be helpful to decide a neoplastic lesion, but absence of p53 does not exclude neoplasia [73]. Several independent studies have already shown that mutation in the p53 gene is in small percentage of cervical tumors [74]. However, a growing body of evidence indicates that other functions of p53 may be equally important to prevent or stall cancer development. During stalling of its function apoptosis terminates but cancer cells proliferates along with mutations. The second protein CK1 is found important for cell functioning but depletion or inhibition of CK1 cancer cells results in reduced colony formation and various type of cell carcinoma. Although, CK1 is highly expressed in tumor tissues, its loss from the cytoplasm is unexpectedly predicted in poor survival [75]. The function of Cdk1 is known as a tumor suppressor protein which is the driving force for mitotic entry, and its activation is tightly regulated by the G2/M checkpoint [76]. Some studies report that, over expression of C53 overrides the G2/M DNA damage checkpoint to promote Cdk1 activation, thereby sensitizing cancer cells to various DNA damage agents [77]. Although, during DNA damage response, activation of checkpoint kinase 1 and 2 (Chk1 and Chk2) is partially inhibited by C53 over expression and thus contributes in tumorigenesis. The next protein is AKT1, whose abnormality leads to defective signaling in many human cancers and mutations in the AKT pathway responsible for cervical cancer [78]. Activation of AKT1 suppresses apoptosis in a transcription-independent manner, which then phosphorylates and inactivates workings machinery of the apoptotic pathway. In many types of cancers, activation or deactivation of multiple oncoproteins, tumor suppressor cell signaling and metabolic regulation intersects the AKT signal transduction pathway [79]. The next protein SUMO1 is detected in some of the cancers and may be used as a predictive marker for prognosis of cancer. A recent study demonstrates a significant increase in protein sumoylation in two leukemic cell lines [80]. The effect can also be measured for cervical cancer. The protein is SUMO2 is also modified variety of cellular proteins leads to the alterations in many signaling pathways associated with their target proteins [81], but its role in cervical cancer is not clear. Further, the protein SRSF1 is up-regulated in cancer and its transcription is activated by the prooncogenic transcription factor Myc. However there is a significant difference in SRSF1 gene copy number that may account for its up-regulation [82]. The next protein XRCC3 acts as DNA repair genes and its function is in the maintenance of integrity of the genetic material, and hence its dysfunction plays critical roles in cancer development [83].

What follows that, all the proteins corresponding to the high degree in disease state have a major contribution in promoting cervical cancer. The proteins having high degree in normal states also have a potential role in some of the cancers and may be present in cervical cancer. Moreover, all the high degree proteins are important for the normal as well as the disease state, but leading to a very different behavior of cell in the two states. In normal cells these proteins are involved in house keeping functions of the cells, where as in disease state these are all found to be either up or down regulated or mutated leading to the disease state.

### Functional properties of proteins having *CC* = 1

Next, we explored the nodes forming complete sub-graphs i.e. proteins having *CC* = 1 in all the networks as they were known to be important for a network. It turned out that for the common networks, there were 64 proteins found which had *CC* = 1 (Table 1). Similarly, for DNC there were 37 proteins (Table 1). Most of the nodes having *CC* = 1 lied towards a very low degree regime. Initially, we considered only the distinctly high degree proteins from the list with *CC* = 1.

From this analysis we found that the proteins having high degree and *CC* = 1 in common and disease states were GSG2, CIT and SRPR, PRR11, HNRNPA0 respectively. We studied the functional role of these structurally significant proteins from the available literatures and experimental studies to understand their biological importance. The first protein, kinase haspin/GSG2 plays an important role in mitosis and protein kinase activity [84]. Over-expression of haspin delays early mitosis [85], which disrupts cohesion binding and sister chromatin association. Manipulation in this pathway results in abnormal proliferation of cells and leads to cancer [86]. Also, down-regulation of CIT (Citron) expression causes knock down of mitotic kinesis and leads to reduction in KIF14 expression, which causes subsequent delay in mitosis, and plays a major role in carcinogenesis [87–89]. Further, SRPR mediates the targeting of nascent secretory and membrane proteins to the rough endoplasmic reticulum and participates in WNT signaling pathway. Any dysregulation in WNT signaling pathways and mutation or alteration in its expression level leads to carcinogenesis causing cervical cancer [90, 91]. Next, PRR11 knock-down causes the dysregulation of multiple critical pathways leading to tumorigenesis and metastasis [92]. Last protein, HNRNPA0 is a substrate of MK-2 and also an interaction partner of the 3′terminal HuR clusters [93]. Over expression of HNRNPA0 contributes to tumorigenesis by stabilizing mRNAs of cytokines and other growth regulators [94, 95]. Cells with decreased HuR have reduced growth and indicate a role for RNA-binding protein in regulating cell proliferation via cyclin mRNA stabilization, further implying that any imbalance in HNRNPA0 may lead to activation of CCND1 protein which is a high degree node in the disease network thereby leading to an increased proliferation and invasiveness of cancer cells. CCND1 is also a downstream target of WNT signaling pathway. So SRPR dysregulation may also lead to the up-regulation of Cyclin D1 via WNT pathway. Thus, these proteins are found either in cervical cancer cell line or anyhow responsible for the occurrence of different types of cancers.

## Discussion

We analyzed the cervical cells for the normal and disease states under the framework of network theory. We investigated the structural properties of protein-protein interaction networks for both the states, using average degree, degree distribution, diameter, betweenness centrality, clustering coefficient and important correlations among these quantities of both the networks. The degree distribution of all the networks is observed to follow power law as found for other biological systems [17]. This indicated that in all the networks there were very few nodes having very high degree. In addition, two power laws were found for the DNC network which made the disease network different from other networks considered here. Also, all the normal and disease networks had very high ⟨*CC*⟩ as observed for other biological networks indicating well connected neighbors. The interesting observation emerged from the nodes having *CC* equal to *one*, reflecting the existence of clique structure in the network. Cliques being considered important as preserved structures during evolution makes the system more robust and stable. The nodes having CC equal to *one* were found to play an important role in occurrence of the disease and may act as potential drug targets.

Further in order to understand changes in the disease states from the normal we generated random controls. The analysis reflected that in contrast to the DNC network, there was enormous deviation in the properties of normal networks from the corresponding configuration model. The interesting observation was that while diameter of all the real networks was much larger than the corresponding random control, the DNC state had diameter very close to that of the random controls indicating a faster signal processing or subtle changes in the proteins that push the normal cells to develop into malignant cells in the disease network. We also analyzed the degree-betweenness centrality correlations of all the real and their corresponding model networks. The overall *k*-*β*_*c*_ correlation for all the networks was positive, but the striking findings of DNC suggested that the value of highest *β*_*c*_ in DNC was as low as that of its corresponding random control. The lower value of *β*_*c*_ of the nodes in the network implicated that they do not come in many pathways. This emphasized that the highest *β*_*c*_ of the node in the network was only due to its degree and no other factors contribute in bringing it into the shortest paths exhibited by other networks. Further, apart from betweenness centrality, there were other properties which can predict the differences in the local behavior of both the normal and disease networks and in order to do so, we analyzed the *k*-*CC* correlation of all the networks.

The *k*-*CC* correlation patterns (Fig 4) depicted that the disease network was less random than the normal network. The DNC networks had comparatively good k-CC correlation indicating modularity. The other networks were found to be deviating from this negative correlation which was interpreted in terms of existence of random interactions in the network. Since, *randomness* has already been emphasized as an essential ingredient for proper functionality of underlying system, [34] disease being less random might be due to the removal of random connections, in turn making disease network less robust.

On the basis of above inferences, we further analyzed the functional properties of high degree proteins in the normal and the disease networks. We found that the proteins which have high degrees in the disease state, were primarily responsible for the occurrence of the disease. The proteins which were high degree in normal states were not found responsible for cervical carcinoma directly but their imbalance may have an important role in changing the normal cells to cervical cancer progression (for eg. TP53 and AKT deregulation) as they exhibited a significant role in other types of cancers.

## Conclusion

To conclude, this framework of studying diseases using network biology and statistical methods provided time efficient, cost effective and a novel approach to understand the complexity of the disease. The analyses also detect structural patterns of the proteins which were responsible for disease onset and progression. The analysis could be useful particularly for those diseases about which much information is unavailable of their genes which are responsible for the occurrence of the disease. The analysis also suggested that instead of targeting a single protein, a group of proteins could be of profound implications and can result in developing novel drug and therapeutic targets. This method could also be extended for analysis of other diseases such as different cancers, neuronal disorders, environmental diseases etc to predict structural and functional aspects of the disease state.

## Materials and Methods

### Data collection and network construction

The network is a set of nodes linked by the edges corresponding to a relations defined between them. In a PPI network, proteins are the nodes and physical and chemical interaction between pair of nodes depicting the links between them. In an attempt to obtain all the proteins of the normal and cervical cancer we got data from various sources i.e., from Uniprot KB (http://www.uniprot.org/) [96], the cervical cancer database (CCDB) (http://crdd.osdd.net/raghava/ccdb/) [97] and Proteome 2D-PAGE Database (http://web.mpiib-berlin.mpg.de/cgi-bin/pdbs/2d-page/extern/index.cgi) [98] in addition to the data available from previous published literatures. To keep the authenticity of data, we considered only those proteins into account which are reviewed and/or cited. We also focussed on most widely studied cervical cancer cell lines whose protein expression data is known in order to add more information. There are several cell lines available but only a few have been exploited for their maximum proteomic insight. Here, we used the data of HeLa cell line [99]. After retrieving the proteins for normal and disease datasets, their interacting partners were downloaded from the STRING database [100].

### Adjacency matrix and structural measures

A number of statistical measures have been proposed to understand specific features of the network [101]. In order to perform such analysis for cervical cancer, we defined the interaction matrix or the adjacency matrix of the network as follows

$$\begin{array}{c} A_{\text{ij}}=\{\begin{array}{c} 1\;\;\text{if}\;i∼j\\ 0\;\;\text{otherwise} \end{array} \end{array}$$ (1)

The most basic structural parameter of a network is the degree of a node (*d*_*i*_), which is defined as the number of neighbors a node has $\left(d_{i}=\sum_{j=1}^{n}A_{ij}\right)$. The degree distribution *P*(*k*) reveals the fraction of vertices’s having degree *k* and is known to be a fingerprint of the underlying network [102]. Another important parameter is the clustering coefficient (CC) of the network, for a node (*CC*_*i*_) which is defined as a ratio of the number of triangles a particular node make with its neighbors and the possible number of triangles a particular node can make. The clustering coefficient of a network can be written as,

$$\begin{array}{c} \left\langle CC\right\rangle =\frac{1}{N}\sum_{i=1}^{N}CC_{i}, \end{array}$$ (2)

The average clustering coefficient of the network characterizes the overall tendency of nodes to form cluster or groups [101]. Further, the betweenness centrality of a node is defined as the fraction of shortest paths between all the pairs of the nodes that pass through that node *i*[32] and can be calculated as,

$$\begin{array}{c} x_{i}=\sum_{st}\frac{n_{st}^{i}}{g_{st}}, \end{array}$$ (3)

where $n_{st}^{i}$ is the number of shortest paths from *s* to *t* nodes that passes through node *i* and *g*_*st*_ is the total number of shortest paths from *s* to *t* in the network. Another parameter is the diameter of the network which measures the longest of the shortest path between all the pairs of the nodes [103].

### Configuration model

We compared the real networks constructed as Eq 1 for the disease and normal states with the corresponding configuration model [28, 104]. To construct a configuration model with degree sequence (*d*_1_,*d*_2_…*d*_*N*_), where *d*_1_ ≥ *d*_2_ ≥ … ≥ *d*_*N*_ was taken to the degree sequence of the corresponding real networks. We created a collection of *N* nodes such that first node had degree *d*_1_, second node had degree *d*_2_, and so on. With equal and uniform probability, two nodes were picked up at random from the collection and they were connected. This process was repeated till no node was left unpicked.
